# The present situation and towards the prevention and control of neurocysticercosis on the tropical island, Bali, Indonesia

**DOI:** 10.1186/s13071-015-0755-z

**Published:** 2015-03-07

**Authors:** Toni Wandra, Kadek Swastika, Nyoman S Dharmawan, Ivan Elisabeth Purba, I Made Sudarmaja, Takahiko Yoshida, Yasuhito Sako, Munehiro Okamoto, Ni Luh Putu Eka Diarthini, Dewa Ayu Agus Sri Laksemi, Tetsuya Yanagida, Minoru Nakao, Akira Ito

**Affiliations:** Sari Mutiara Indonesia University, Medan, North Sumatra Indonesia; Department of Parasitology, Faculty of Medicine, University of Udayana, Denpasar, Bali Indonesia; Department of Veterinary Parasitology, Faculty of Veterinary Medicine, University of Udayana, Denpasar, Bali Indonesia; Department of Health Science, Asahikawa Medical University, Asahikawa, Hokkaido Japan; Department of Parasitology, Asahikawa Medical University, Asahikawa, Hokkaido Japan; Section of Wildlife Diversity, Center for Human Evolution Modeling Research, Primate Research Institute, Kyoto University, Inuyama, Aichi Japan; Laboratory of Veterinary Parasitology, Joint Faculty of Veterinary Medicine, Yamaguchi University, Yoshida, Yamaguchi Japan

**Keywords:** Neurocysticercosis, Cysticercosis, Taeniases, Bali, Indonesia, Asia, Prevention, *Taenia solium*, *Taenia saginata*, *Taenia asiatica*, Parasitic zoonosis, Soil transmitted helminthiases

## Abstract

Neurocysticercosis (NCC), which is caused by accidental ingestion of eggs of the pork tapeworm, *Taenia solium*, was common in Bali, Indonesia until the early 1990s. However, improved education on hygiene and sanitation, a move to keeping pigs indoors, and improvement of economic and living conditions have substantially reduced the occurrence of NCC in Bali. Since 2011, *T. solium* tapeworm carriers (*T. solium* taeniasis) and heavily infected pigs and dogs have exclusively been detected from villages in mountainous regions of northeastern Bali where NCC and ocular cysticercosis (OCC) cases have also been identified. In response to this continued area of high infection, a one-day workshop was convened to discuss how to prevent and control this potentially lethal zoonotic parasitic infection in Bali. This review presents an overview of the current status of *T. solium* taeniasis and cysticercosis in Indonesia and proposes a strategy for the prevention and control of this zoonosis in Bali.

## Introduction

*Taenia solium* (also known as the pork tapeworm) and *Taenia saginata* (also known as the beef tapeworm) are human cestodes with a cosmopolitan distribution. These cestode infections result in both economic and public health impacts on affected communities [1-24]. Cysticercosis, which is caused by the larval stage of *T. solium*, is prevalent in humans and pigs mainly in many developing countries of the Americas, Africa and Asia but also in Europe as well [1-3,14,18,19,21,22,24]. Approximately 50 million people are suffering from neurocysticercosis (NCC) due to *T. solium* globally and more than 50,000 deaths per year are due to NCC [6,25]. Increased international travel and immigration are resulting in NCC being diagnosed and treated more frequently in non-endemic areas [5,26-33].

**Figure 1:**
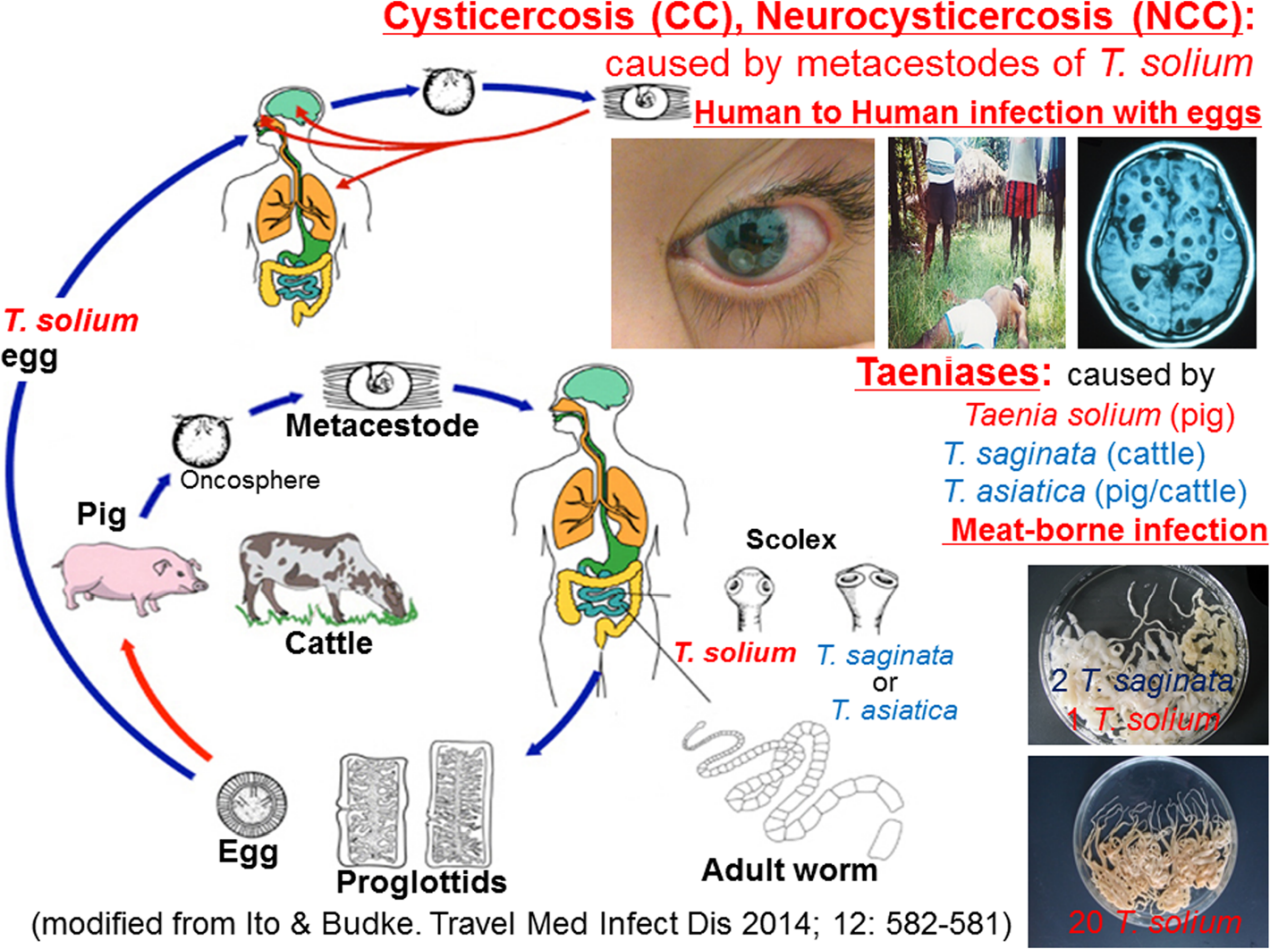
**The life cycle of three human** ***Taenia*** **tapeworms (modified from CDC** [22]). Photos of ocular cysticercosis, epileptic attack in Papua (=Irian Jaya), neurocysticercosis, a case of dual infection with two *T. saginata* and one *T. solium* tapeworms, and a case of 20 *T. solium* tapeworms are from Swastika *et al.* [90], Wandra, unpublished, Ito *et al*. [76], Li *et al.* [49] and Ito *et al.* [109], respectively.

*T. solium* completes its life cycle using pig intermediate hosts and human definitive hosts (Figure 1). However, dogs can also become infected with cysticerci through the ingestion of parasite eggs [34]. Although it is conceived that humans were only obligatory intermediate host when this parasite emerged as a human parasite without involvement of pigs, humans are now paratenic hosts unless cannibalisms happen under extremely chaotic unusual conditions [35,36]. In humans, NCC resulting in epileptic seizures can be a life-threatening disease [7,12,16,25]. Taeniasis occurs through eating uncooked or undercooked pork contaminated with cysticerci, the metacestode stage of *T. solium*. Adult tapeworms then mature in the intestines of the infected person. Eggs from mature adult worms can infect pigs, dogs and humans resulting in cysticercosis. While ingesting infected pork is the most common way for a person to acquire *T. solium* taeniasis, eating dog meat does occur in parts of Asia including Indonesia, and may be responsible for some cases [34,36,37].

This review presents an overview of the current status of human taeniases caused by *T. solium*, *T. saginata* and *Taenia asiatica* and cysticercoses caused by *T. solium* (humans and livestock) and *Taenia hydatigena* (livestock) in Indonesia. A strategy for the prevention and control of this zoonosis in Bali is also proposed. Presented information is based on an ongoing joint project towards the control of human NCC in Indonesia which started in 1996 using several Japanese research funds. The topics addressed in this review are based on summaries from a one-day international workshop entitled “Strengthening of Prevention and Control of Taeniasis/Cysticercosis and Soil Transmitted Helminthiases in Bali, Indonesia” held at the Faculty of Medicine, University of Udayana, Bali on 22 September 2014.

## Review

### Taeniases caused by three human *Taenia* species in Indonesia

**Figure 2:**
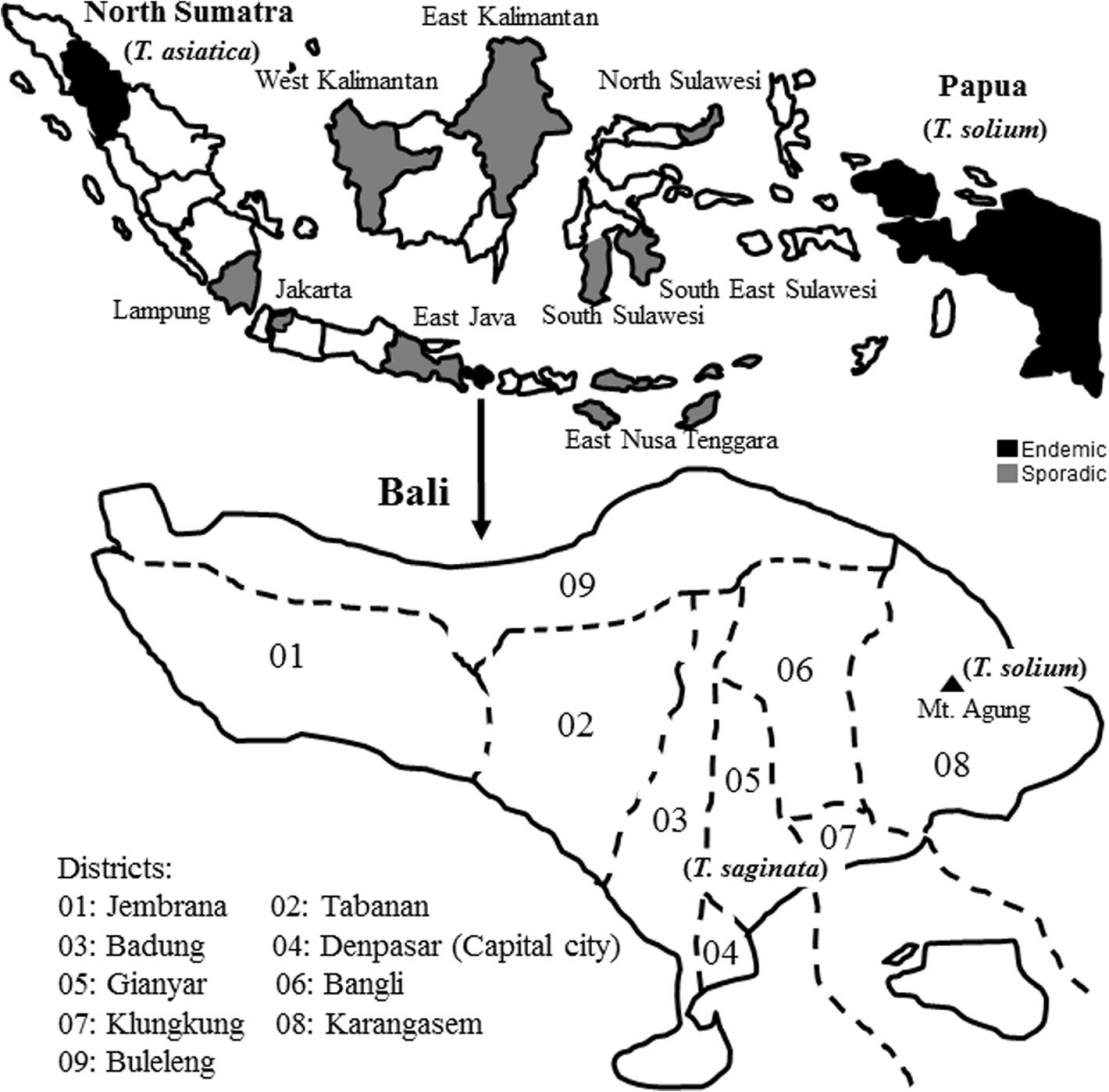
**Geographic maps of Indonesia (upper) showing endemic areas of three human** ***Taenia*** **species (** ***T. asiatica*** **, endemic in North Sumatra;** ***T. saginata*** **and** ***T. solium*** **, endemic in Bali; and** ***T. solium*** **, endemic in Papua) and Bali (lower) (from** [38-43]).

Three human *Taenia* species have been confirmed in Indonesia: *T. solium* has been reported mainly from Papua and Bali*, T. saginata* from Bali, and *T. asiatica* from Samosir island, North Sumatra [38,39]. The distribution of these parasites is not well documented in many Indonesian provinces. However, taeniasis and NCC have been reported from Lampung, Jakarta, East Jawa, East Nusa Tenggara, West Kalimantan, East Kalimantan, North Sulawesi, South Sulawesi, and South East Sulawesi (Figure 2) [38-43]. There is the concern that tapeworm carriers may establish new life cycles via traveling to different islands. For example, if a *T. asiatica* carrier from Samosir Island were to visit Bali, there is the chance of establishing the *T. asiatica* life cycle in Bali. Variations in parasite distribution may also be due to cultural differences among the endemic areas (islands) [44,45]. For example, the Batak people in Samosir eat uncooked viscera of pigs but eat cooked pork, “*sang-sang*”, whereas people in Bali eat uncooked pork “*lawar*” but do not eat uncooked viscera of pigs [43,46,47]. So, *T. asiatica* is still common in Samosir, whereas *T. solium* was common in Bali until the early 1990s, since cysticerci of *T. asiatica* develop in the viscera of pigs but not in meat.

In Thailand [48] and China [49] where *T. saginata*, *T. asiatica*, and *T. solium* are sympatrically distributed, hybrids or hybrid-derived tapeworms of *T. saginata* and *T. asiatica* have been confirmed by mitochondrial and nuclear DNA analyses [50-52]. Therefore, *T. saginata* and *T. asiatica* specimens collected in Indonesia should also be analyzed to determine whether or not hybridization is occurring.

## Cysticercosis in Indonesia

### *Papua (Irian Jaya):* T. solium *taeniasis and NCC highly endemic*

**Figure 3:**
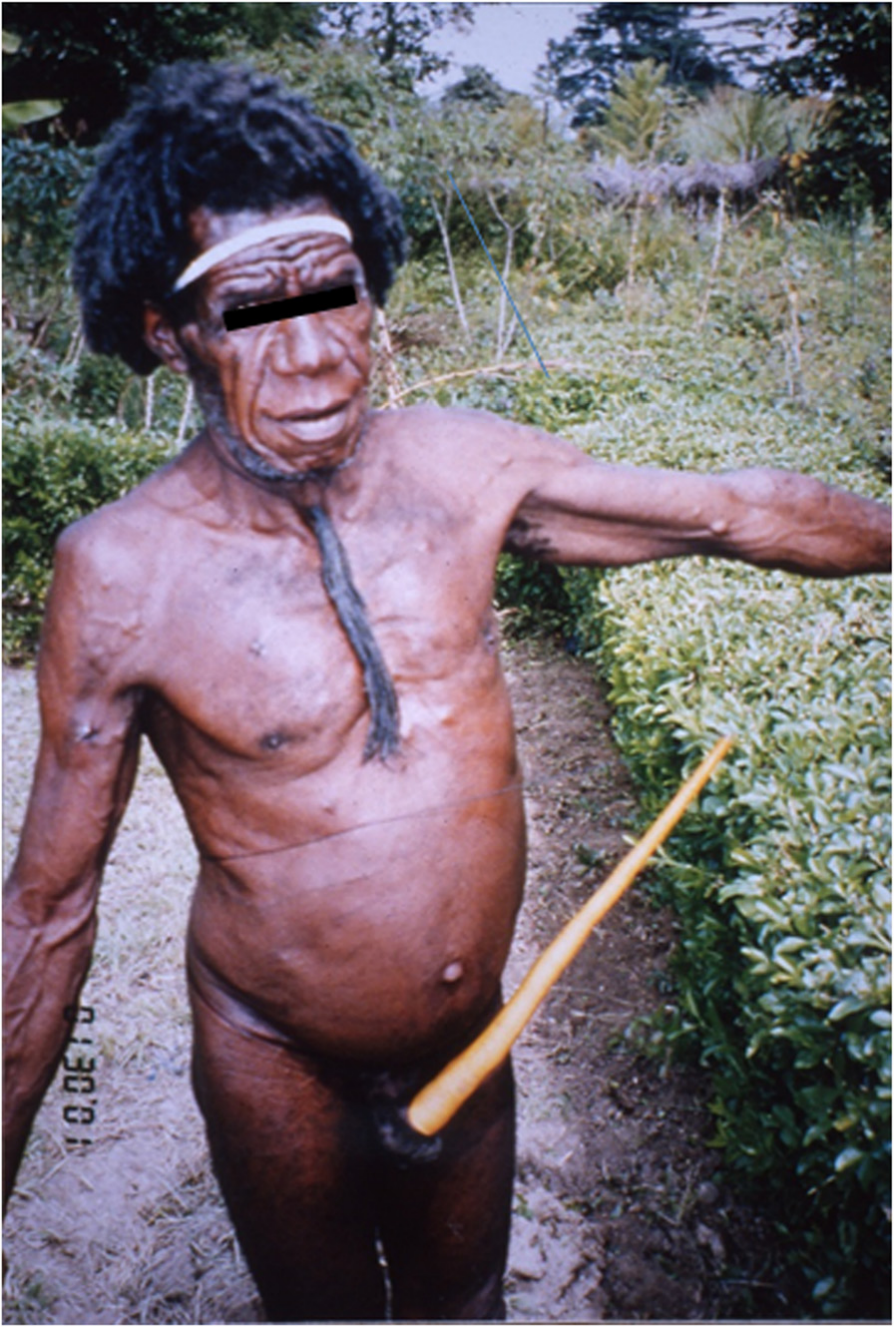
**Subcutaneous cysticercosis patient in Papua.** This patient was often attacked by epileptic seizures [41,43,58-63].

Papua (formerly Irian Jaya) is a known high endemic area for NCC [39,53-65]. Outbreaks of NCC in Papua had been reported since the early 1970s, with historical reports briefly reviewed by Simanjuntak *et al*. [38]. The Indonesian Government started a 10-year project to control taeniasis and NCC in Papua from 1990 [39,41,53-65]. Recent studies have revealed that Papua is still highly endemic with most of the NCC cases also presenting with subcutaneous cysticercosis (Figure 3) [41,43,58-63]. Serology is important for the early diagnosis and treatment of asymptomatic NCC cases as well as to identify infected pigs and dogs [34,41,61], and remove them from the food chain. Results from a commercially available ELISA [66] without any direct evidence of infection should be re-evaluated by immunoblot using purified antigens [67-78], since cross-reactions may occur among the various *Taenia* spp. that infect pigs (see below) [36,37]. Optimally, serological tools should be evaluated against pig necropsy findings as is currently underway in Karangasem, Bali (Dharmawan *et al*. unpublished).

### *Bali:* T. saginata *and* T. solium *taeniases and NCC*

**Figure 4:**
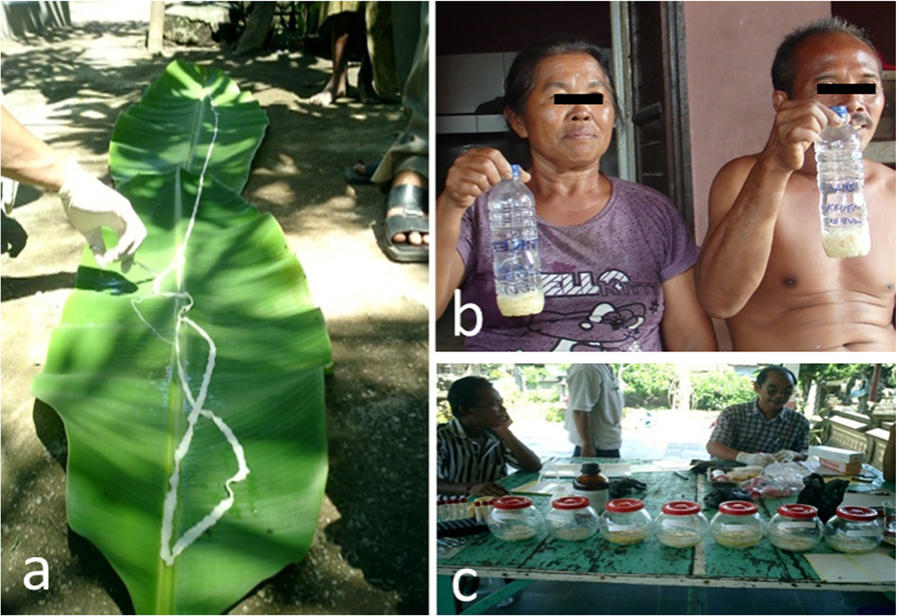
***T. saginata*** **(a, b, c) expelled after anamnesis and microscopy in Gianyar.** One long *T. saginata* tapeworm expelled from one tapeworm carrier **(a)**, *T. saginata* tapeworms expelled from wife and husband and kept in bottles **(b)**, and *T. saginata* tapeworms expelled from 8 carriers during one day survey **(c)**.

Prior to the early 1990s, NCC was considered a fairly common disease in Bali [41,79-91]. However, health education programmes have dramatically reduced the number of cases in the last twenty-five years. NCC cases were reported sporadically and *T. solium* taeniasis cases had not been detected via field surveys since 2002 [43,46,47,68,89]: From 2002 until 2010, all *Taenia* tapeworms collected through epidemiological studies were identified as *T. saginata*, with the majority of infections occurring in the district of Gianyar (Tables 1 and 2, Figures 2 and 4). Detection of taeniasis carriers was based on questionnaire and stool examination for eggs of *Taenia* and soil transmitted helminths (STHs). Eggs of *Ascaris*, *Trichuris*, and hookworms are more common everywhere in Bali (Wandra *et al*. unpublished). Therefore, the project on taeniasis is one part of STH surveys with medication of STH carriers.

**Table 1:** **Summarized data of taeniasis cases and seroprevalence of cysticercosis by district in Bali, 2002–2014** [40,43,47,88,89]

**District (Year)**	**No. of** ***T. saginata*** **taeniasis cases**	**No. of** ***T. solium*** **taeniasis cases**	**Seroprevalence of cysticercosis in humans (%)**	**Seroprevalence of cysticercosis in pigs (%)**
Gianyar (2002)	32	-	0.8 (1/125)	NA
Gianyar (2004)	14	-	0.0 (0/46)	NA
Gianyar (2005)	5	-	0.0 (0/13)	NA
Gianyar (2006)	2	-	0.0 (0/39)	NA
Gianyar (2007)	3	-	4.2 (1/24)	NA
Gianyar (2008)	4	-	NA	NA
Gianyar (2009)	7	-	NA	NA
Gianyar (2010)	18	-	0.0 (0/24)	NA
Gianyar (2011)	9	-	5.4 (8/147)	NA
Gianyar (Jan 2013)	6	-	0.1 (1/13)	NA
Gianyar (Sept 2013)	9	-	7.1 (1/14)	NA
Gianyar (2014)	4	-	NA	NA
Badung (2004)	1	-	0.0 (0/91)	NA
Denpasar (2004)	9	-	0.0 (0/49)	NA
Denpasar (2005)	2	-	0.0 (0/16)	NA
Denpasar (2010)	3	-	0.0 (0/54)	NA
Karangasem (*urban area*, 2006)	1	-	2.8 (1/36)	NA
Bangli (2007)	0	-	0.0 (0/32)	NA
Tabanan (2008)	0	-	0.0 (0/42)	NA
Jembrana (2008)	0	-	0.0 (0/84)	NA
Klungkung (2009)	0	-	0.0 (0/100)	NA
Buleleng (2009)	0	-	0.0 (0/47)	NA
Karangasem (*rural area*, 2011)	-	3	6.3 (11/175)	11.6 (5/64)
Karangasem (Jan. 2013)	-	6	5.1 (11/214)	18.0 (31/164)
Karangasem (Sept. 2013)	-	2*	4.2(5/118)*	6.9 (7/101)*
Karangasem (2014)	-	2*	#	#
Total	129	13	2.6 (38/1489)	13.1 (43/329)

NA: no data available.

^#^in confirmation.

*Swastika et al. unpublished.

**Table 2:** **Neurocysticercosis (NCC) in Gianyar district, Bali, 2003, 2007, and 2010** [40,43,47,88,89]

**Area/Hospital (year)**	**Diagnose**	**No. of case**
Gianyar/Sanglah Hosp. (2003)	NCC	1 (disseminated cysticercosis)
Gianyar (2007)	NCC (dual infection)	1 (with *T. saginata* taeniasis)
Gianyar (2010)	NCC(1)/cysticercosis (2)	3 (with *T. saginata* taeniasis) (dual infection)

When tapeworm carriers in Gianyar were treated with praziquantel (PZQ) (15 mg/kg BW), one individual had an epileptic seizure within half a day of receiving the drug. This patient was later confirmed as having previously asymptomatic NCC, which became symptomatic after receiving PZQ [89,92]. This case was also confirmed serologically to be cysticercosis [89]. If a lower dose of PZQ (5 mg/kg BW) which is sufficient for expulsion of tapeworms but not sufficient for damaging cysticerci was administered [92], this veiled asymptomatic NCC could not become symptomatic, and we could not find dual infections with *T. saginata* taeniasis and *T. solium* NCC. It is believed that *T. saginata* infections in people were due to consumption of undercooked beef “*lawar*” contaminated with cysticerci. In contrast, it is believed that the NCC case was due to accidental ingestion of *T. solium* eggs from a tapeworm carrier who had been infected in a *T. solium* endemic area of Bali.

### *Bali (eastern slope of Mt. Agung in Karangasem):* T. solium *tapeworm carriers and infected pigs*

**Figure 5:**
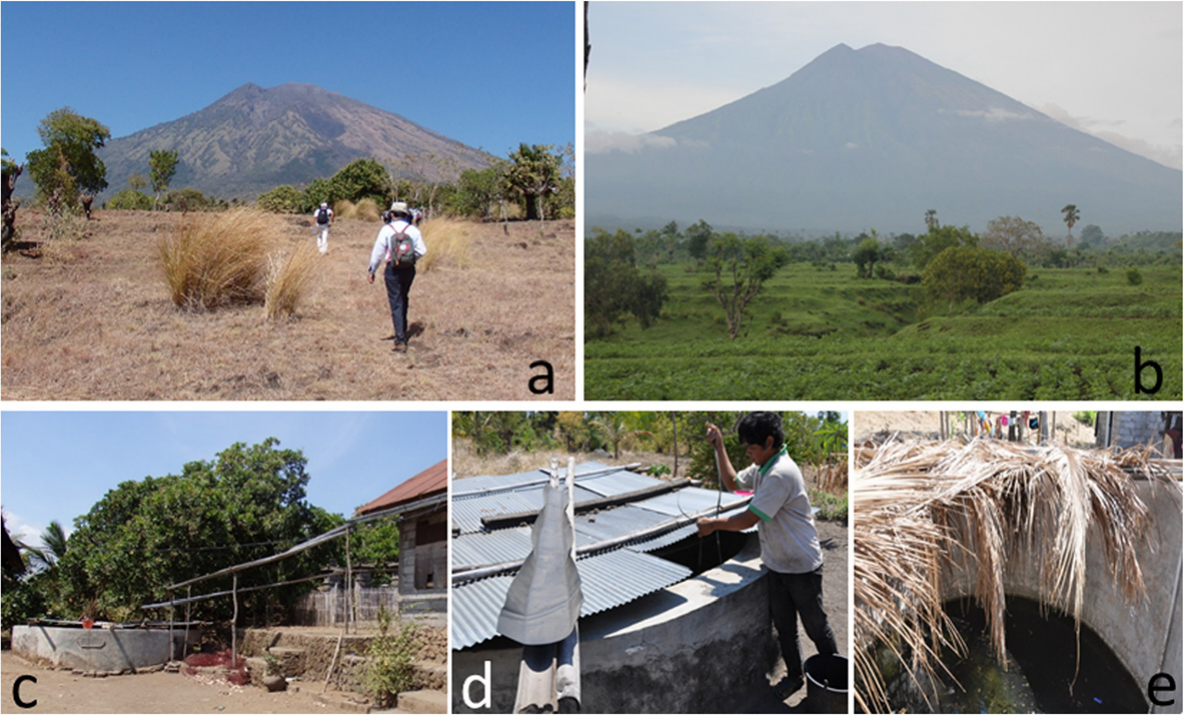
**Landscape of the eastern slope of Mt. Agung (a, b) and water tanks for dry season (c, d, e).** Dry season in September 2014 **(a)** and rainy season in January 2012 **(b)**. Right half of the mountain **(a)**: the eastern slope is completely brown. Southern slope in the left half is better in green, Western slope is completely green for all seasons (not shown).

In December 2010, a case of ocular cysticercosis (OCC) was confirmed in a 9-year-old girl (Figure 1) from a remote village in the Kubu sub-district of Karangasem (Figure 5) [90]. The village is located on the eastern slope of Bali’s highest mountain, Mt. Agung (altitude 3,132 m). In highly endemic areas in other countries, it is not so easy to detect tapeworm carriers as found in this small area (Table 1) [11,93-96]. A field survey carried out in January 2011 in the patient’s village and neighbouring villages revealed three individuals with *T. solium* taeniasis (Table 1) [90,93]. Two cases (a 4-year-old girl and a 38-year-old man) were from the same village where the original OCC case was identified. The third case (35-year-old woman) was from a neighbouring village. Table 1 summarizes human taeniasis prevalence data collected in Karangasem (Kubu sub-district) from January 2011 to September 2014 and Gianyar (Sukawati sub-district) from 2002 to 2014. In total, six *T. solium* taeniasis cases (6/265, 2.26%) were detected in 2013, and additional two cases (2/138, 1.45%) were identified in 2014 in Karangasem (Swastika *et al*. unpublished).

**Figure 6:**
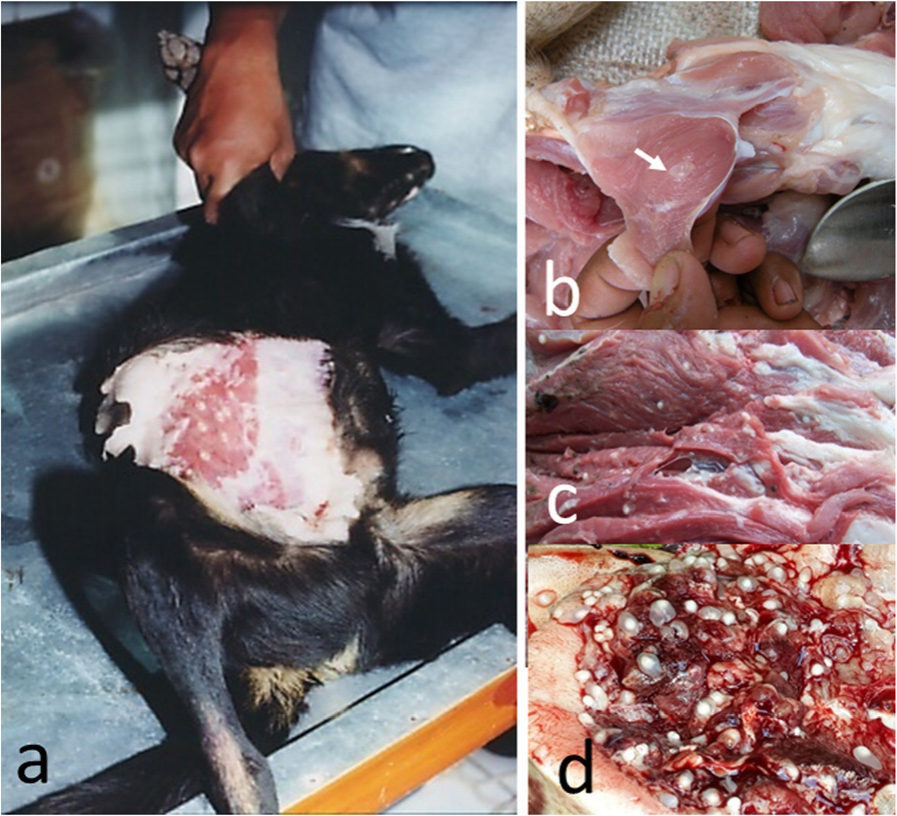
**Cysticerci of** ***T. solium*** **in one dog (a) and three pigs (b, c, d).** One dog full of cysticerci **(a)** [34] and pigs contaminated with light **(b)**, middle **(c)** and heavy **(d)** infections with cysticerci confirmed based on the naked eye-ELISA (Dharmawan *et al.* unpublished) [36,67,69,70].

In addition to human taeniasis cases, pigs and dogs with cysticerci have been found in this region (Figure 6). Thus far, all tapeworms expelled in Gianyar have been confirmed to be *T. saginata* by molecular analysis. In contrast, all tapeworms except for one expelled in villages in the Kubu sub-district have been *T. solium.* The one *T. saginata* specimen was obtained from an individual living in a more urban village and is speculated to have been an imported case from Gianyar. The fact that taeniasis cases due to *T. solium* have, thus far, all been exclusively from mountainous villages strongly suggests that contaminated pork is being consumed within these villages, but is not being sold to other regions. It strongly suggests that *T. solium* is still exclusively spreading in relatively small rural and remote mountainous villages in Karangasem.

The weather on the eastern slope of Mt. Agung differs from other areas in Bali, with little grass available during the dry season (Figure 5). Since all climbing routes to Mt. Agung are located on the western side of the mountain, the endemic villages are almost completely isolated. However, residents of this area often go to the local capital city of Denpasar or other districts to find work, which has the potential to result in an outbreak of NCC in these non-endemic areas [85,97-100].

### Modern tools for identification of taeniasis carriers, cysticercosis patients, and cysticercosis in pigs

#### Taeniasis

Multiplex PCR was first used to identify parasites that were expelled after chemotherapeutic therapy, since the expelled tapeworms were often damaged and without a scolex, which is useful for the differentiation of *T. solium* with hooklets on the scolex from two other *T. saginata*-like species without hooklets. As eggs of *Taenia* spp. are impossible to be identified morphologically, molecular analysis using even a single egg in feces [101] is essential for the identification of the *Taenia* species. While multiplex PCR was useful for differentiation of adult worms [75,76,97-100], it was not optimal for the detection of DNA in stool samples. It is important to be able to detect parasite-specific DNA in stool samples to avoid inducing epileptic seizures in taeniasis cases that also have asymptomatic NCC [88,89]. Recently, loop mediated isothermal amplification (LAMP) and copro-LAMP have been utilized to obtain real-time identifications of *Taenia* species [102-104]. Molecular identification using a haplotype network of mitochondrial gene(s) is another useful tool for identifying the infecting species [105,106].

#### Cysticercoses in pigs and dogs

Pigs confirmed to be naturally infected with *T. solium* show antibody responses to antigens purified by preparative iso-electric focusing [69,70] and recombinant antigens [67,71,107,108]. Similar results are also achieved using a more simple and cheap, cathion-exchange chromatography [67]. Antibody responses in pigs in endemic areas have been tested using an ELISA with tests read by looking for a colour change with the naked eye (Dharmawan *et al.* unpublished) [36,37]. The majority of pigs showing strong positive responses were confirmed to be infected with *T. solium* cysticerci, with the remainder infected with *T. hydatigena.* Pigs infected with *T. hydatigena* tended to have a much weaker positive test than pigs infected with *T. solium* (Dharmawan *et al.* unpublished). Cysticercoses in pigs co-infected with these two species should also be identified due to the risk of human NCC [70,109].

Available serology is also applicable to dogs in endemic areas [34]. Residents of villages in Kubu are known to eat dog meat, with local dogs confirmed to be infected with *T. solium.* Therefore, studies are needed to evaluate the role of dogs in the *T. solium* life cycle. In addition, the role of dogs should also be included in local education programmes [34].

### International meetings on NCC in Bali

NCC was discussed at two International meetings on “Recent Progress in Parasitology” (August 2007) and “Neurological Diseases” (November 2009) held in Denpasar. The meetings were aimed at both clinicians and medical researchers. These meetings were in addition to a symposium on cestode zoonoses in Asia which has been held almost every year since 2000 in Thailand, and in Japan (2006), and in Korea (2007) [39,60,64,68,72,73,76-78,89,98,99,110-112]. On 22 September 2014, a workshop focusing exclusively on the control of taeniasis and NCC was held at the University of Udayana. The meeting was aimed at personnel working in medicine, veterinary medicine, public health, meat inspection, and the local government.

### Towards control of NCC

Since 2011, *T. solium* in Bali has been maintained in a small area on the eastern slope of Mt. Agung. However, the prevalence and distribution of NCC seems to be increasing through immigration of individuals from this endemic area to the Denpasar metropolitan area. As shown in Figure 5, during the dry season, villagers have difficulty in obtaining safe drinking water and feed for their pigs. Therefore, pigs are often left to roam and scavenge during the dry season. In addition, during the dry season, local inhabitants often go to Denpasar or other larger cities to find work, increasing the risk of bringing *T. solium* to currently non-endemic locations. This phenomenon has also been seen in refugee villages along the Thailand-Myanmar border [48], and in Tibetan communities in Sichuan Province, China [49].

Due to the stigma attached to being a tapeworm carrier, most people are not willing to submit a stool sample for evaluation. It is also difficult to change local food consumption behavior *lawar,* a traditional food made with uncooked beef or pork is commonly consumed in the endemic areas of Bali. In Gianyar district, while the majority of people stop eating *lawar* after being diagnosed with tapeworm(s), it is not uncommon for these same individuals to start eating *lawar* again 1–3 months later (Figure 2) [46]. Based on questionnaires administered to residents of a *T. solium* endemic area in Karangasem district, 29% (18/62) of families have no sanitary facilities and people defecate in the garden, 83.9% (40/46) of pig owners keep their pigs in a fenced field, 10.9% (5/46) keep their pigs in an open common pasture, and 2.2% (1/46) allow their pigs to roam free [43]. Based on direct observations, environmental sanitation and personal hygiene is also very poor in this region.

In Bali, infection with *T. saginata* is believed to be related to consumption of beef *lawar.* Quality control of beef and pork is difficult due to the presence of illegal slaughterhouses in addition to a limited number of official meat inspectors. In study conducted in 2002–2004, three of 56 identified tapeworm carriers were *lawar* sellers who acknowledged suffering from *T. saginata* taeniasis for 1–10 years. Several other taeniasis carriers indicated that they bought *lawar* from these sellers (Wandra, personal communication).

The health sector budget allocated by the central government for taeniasis control is very limited due to the presence of other important communicable diseases and the need for resources to be channeled into the country’s environmental health programme. There is also very little funding to pursue control measures at the provincial and district levels. Similarly, *T. solium* has a low priority for the agricultural sector and, therefore, receives little to no funding from this sector.

### Opportunities

Approximately more or less than 3 million travelers visiting the island of Bali every year may be at risk for acquiring *T. solium* cysticercosis. Fortunately, the identified highly endemic areas are not commonly visited by tourists. Due to the limited geographic distribution of the parasite, it may be possible to control and eradicate *T. solium* from Bali given the appropriate resources. Successful control of *T. solium* transmission on Bali could then be used as a model for other islands in Indonesia and beyond.

## Recommendations

Based on recommendations put forth during the 2014 workshop and taking into consideration local cultural practices, the typical education level of local inhabitants, socio-economics, sanitation and personal hygiene conditions in the endemic area of Bali, the following prevention and control activities were recommended.

1. Combine the prevention and control of taeniasis and NCC with the prevention and control of STHs. As STHs including *Ascaris*, *Trichuris* and hookworms etc. are more common than *Taenia*, detection of taeniasis carriers are one part of STH surveys. All STH carriers have been medicated through all taeniasis projects in Indonesia.
2. Review and strengthen the ‘legal aspects’ for the prevention and control of taeniasis and NCC at the provincial, district, and local level. This would include standardizing policies and methods for the distribution of guidelines for the prevention and control of taeniasis and NCC. It would also include better law enforcement to prevent the occurrence of illegal slaughterhouses.
3. Strengthen buy-in of policy makers, stake holders, professional organizations, universities, NGOs, and members of the private sector.
4. Intensify active and passive surveillance, with prompt treatment of identified tapeworm carriers.
5. Conduct periodic health inspection of *lawar* sellers and their family members.
6. Conduct health investigations of family members and neighbours of newly diagnosed NCC patients.
7. Improve public health education focusing on personal hygiene, environmental sanitation, and practices related to pig and cattle rearing, with an emphasis on primary school-aged children.
8. Develop and distribute IEC media on taeniasis and NCC prevention that has been translated into the local languages/dialects and edited to be socially and culturally appropriate.
9. Further invest in local health education programmes by training additional health workers and then having them participate in a train-the-trainer programme.
10. Strengthen the epidemiological surveillance of taeniasis and NCC in Bali by using validated diagnostic tools.
11. Provide additional funding for the prevention and control of taeniasis and NCC.
12. Improve the meat inspection system and conduct studies to determine the prevalence and distribution of infected animals.
13. Encourage political commitment and inter-sectoral collaboration at the local, national, and international levels.
14. Put in place a system to monitor and evaluate the taeniasis and NCC prevention and control programme in Bali.

Vaccination of pigs is an additional highly promising action plan [113-117]. Detection of pigs contaminated with cysticerci of *T. solium* by simple but reliable serology (Dharmawan *et al.* unpublished) [36,37,67,70], and simultaneous vaccinations of all pigs by oral treatment with oxfendazole (30 mg/kg) at the same time as the booster vaccination [116,117] may be the best. However, through sustainable education which almost succeeded in control of taeniasis and NCC in Bali over the past two decades, further sustainable education and improvement of living environment and treatment of *T. solium* taeniasis carriers, especially in endemic area, Karangasem, may be sufficient for successful control of taeniasis and NCC in Bali. If the budget for the control of NCC is big enough to introduce vaccination trials, it is the best.

In order to strengthen the NCC prevention and control programme in Bali, a Research Center at University of Udayana will be established. University of Udayana was selected due to its hospital’s clinical experience with the treatment of NCC as well at the presence of faculties of medicine and veterinary medicine. This new research center will complement the current work being conducted on the prevention and control of other neglected tropical diseases, i.e. rabies in Bali.

## Conclusions

An overview of the current status of *T. solium* taeniasis and cysticercosis in Indonesia reveals the importance of a strategy for the prevention and control of this zoonosis in Bali.
